# Emphysematous Pyelonephritis Complicated by Necrotising Fasciitis and Massive Pulmonary Embolus: A Regional Australian Experience

**DOI:** 10.7759/cureus.18347

**Published:** 2021-09-28

**Authors:** Joshua Teo, Munasinghe T Silva, Henk Van Rooyen

**Affiliations:** 1 Surgery, Queensland Health, Brisbane, AUS; 2 Surgery, Queensland Health, Hervey Bay, AUS

**Keywords:** obstructive pyelonephritis, fournier gangrene, staghorn, regional hospital, emphysematous pyelonephritis, necrotising fasciitis

## Abstract

A 39-year-old female presented to a regional Australian hospital with diabetic ketoacidosis. Urine microscopy, culture and sensitivity (MCS) on arrival revealed 500 leukocytes and eventually culture grew pansensitive E. coli. Patient was transferred to ICU for ongoing care where she remained tachycardic despite resolution of her diabetic ketoacidosis. A CT pulmonary angiogram was performed which found a right lower lobe pulmonary embolus for which therapeutic anticoagulation was commenced. However, tachycardia persisted and the patient became febrile on day three of admission. A CT abdomen pelvis was performed which revealed left-sided emphysematous pyelonephritis secondary to a large staghorn calculus. Significant subcutaneous emphysema was also found in the left flank. A general surgery review was requested and the case was discussed with the urology team located at a tertiary centre. The patient was subsequently transferred to a tertiary hospital under urology where she underwent a left nephrectomy and wound debridement. This was complicated by colonic perforation and was repaired with an omental patch with a loop ileostomy formed. Patient underwent a total of six relooks and debridements before the wound was closed with a combination of delayed primary closure and split-thickness skin graft.

## Introduction

Necrotising fasciitis is a condition that confers high rates of morbidity and mortality. Most common causes include cellulitis from insect bites, intravenous drug use, trauma, and previous operations. In this case report, we discuss the rare entity of emphysematous pyelonephritis as the underlying pathology causing necrotising fasciitis. We also discuss the challenges associated with delivering care to unwell patients in regional Australia.

## Case presentation

A 39-year-old female presented to a regional Australian hospital with a three-day history of polydipsia, polyuria and fatigue. This was on a background of a previous stroke suffered 11 years ago, resulting in left-sided hemiplegia and epilepsy. Patient was not a known diabetic. 

Vital signs on initial presentation included tachycardia to 160 beats per minute and mild hypotension of 110/80 mmHg. Abdomen was soft non-tender. Patient also had a strong ketotic breath. Venous blood gas was done which showed a pH of 7.36, lactate of 2.5, blood glucose of 44, and ketones of 6.5. Urine microscopy was also performed which showed 500 leukocytes, 80 erythrocytes and 10 epithelial cells. Impression of the patient at the time of admission was that the patient’s urinary tract infection had unmasked undiagnosed diabetes mellitus in the form of diabetic ketoacidosis (DKA). 

Patient was commenced on intravenous fluid resuscitation, insulin infusion and intravenous antibiotics (piperacillin-tazobactum and gentamycin). Patient was admitted to the intensive care unit for ongoing care. 

**Figure 1:**
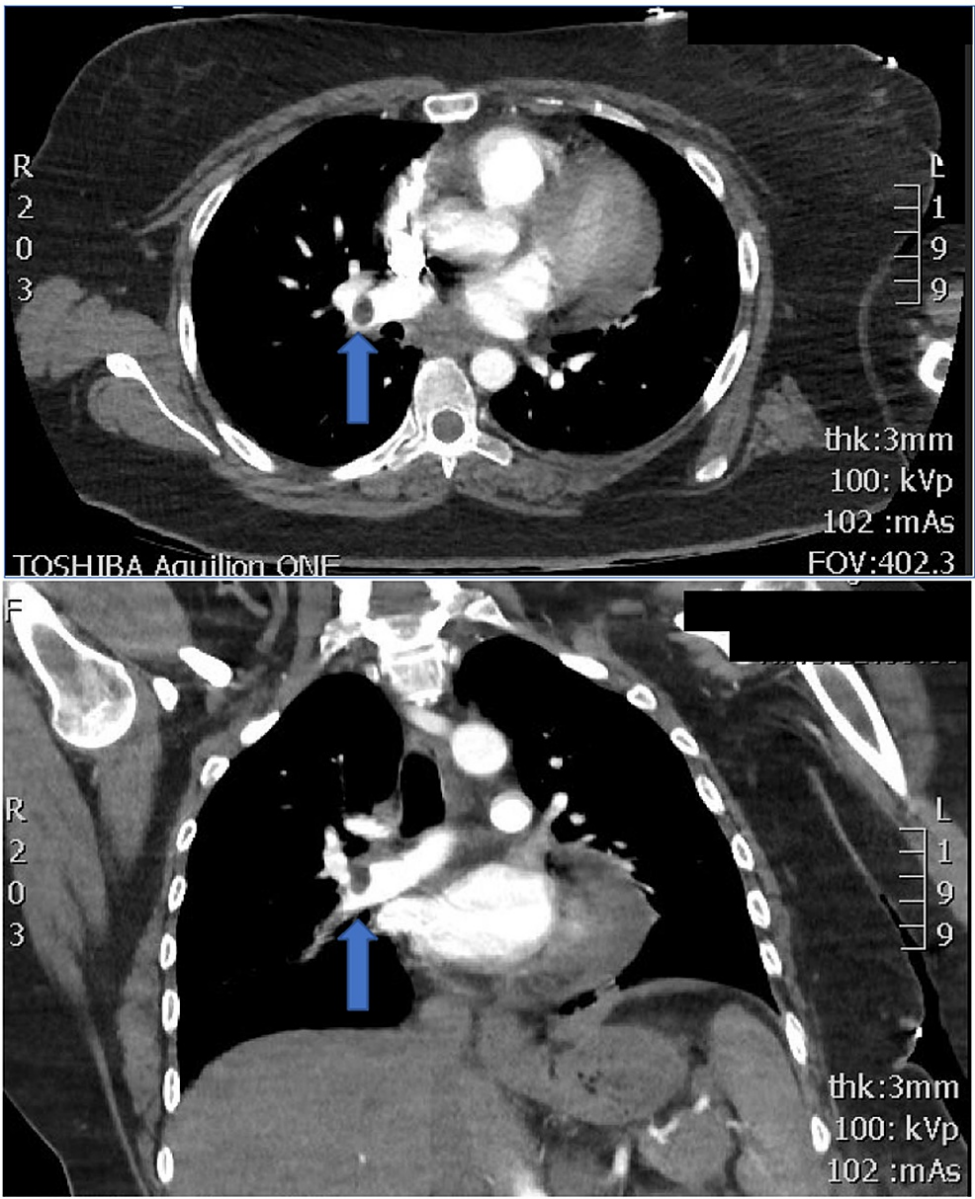
CT pulmonary angiogram - right lower lobe pulmonary embolus.

During the patient’s stay in intensive care, patient’s diabetic ketoacidosis resolved and insulin infusion was ceased on day 2 of admission. However, the patient remained tachycardiac to 116 beats per minute with an oxygen requirement of 4 litres on nasal prongs so a CT pulmonary angiogram was performed. An occlusive thrombus was found in the right lower lobar artery with CT evidence of right heart strain. Patient was then commenced on therapeutic anticoagulation (Figure 1). 

**Figure 2:**
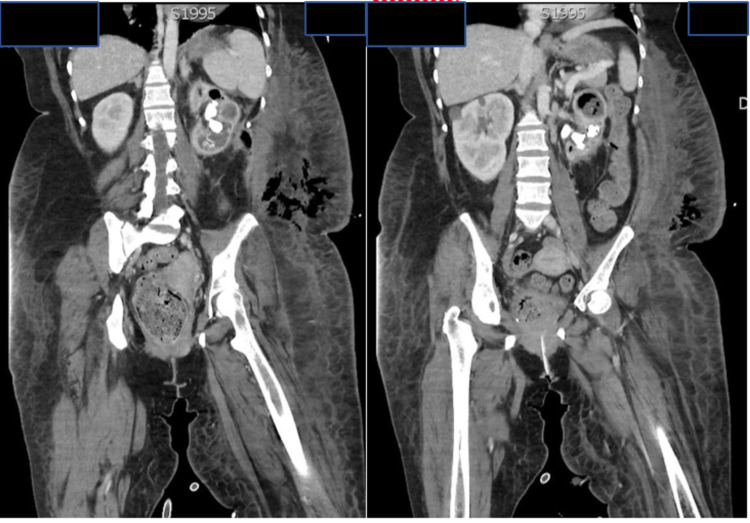
CT abdomen pelvis: large left staghorn calculus with obstructed infected left kidney with emphysematous left pyelonephritis with soft tissue gas in the left retroperitoneum and left flank/posterolateral soft tissues in keeping with necrotising fasciitis.

On day 4 of admission, patient remained tachycardic despite therapeutic anticoagulation and then became febrile. A computed tomography (CT) scan of abdomen and pelvis was done to rule out pyelonephritis given the known urinary tract infection (Figure 2). CT found a large left staghorn calculus with an obstructed infected left kidney. An additional finding included emphysematous left pyelonephritis with soft tissue gas in the left retroperitoneum and left flank/posterolateral soft tissues in keeping with necrotising fasciitis. 

**Figure 3:**
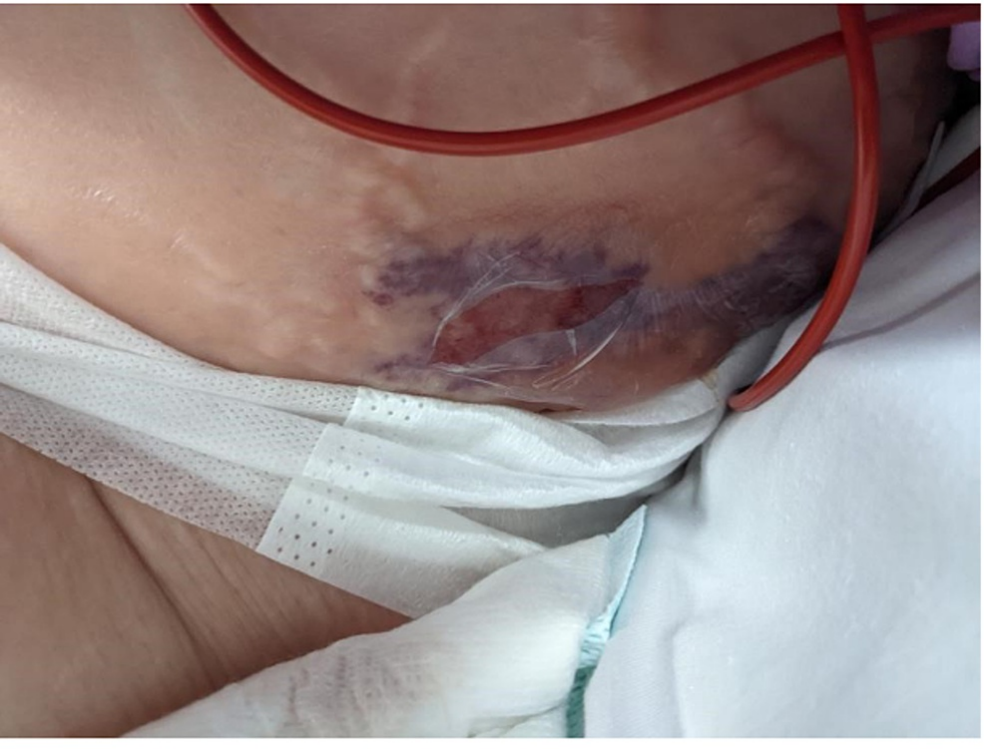
Clinical photo of patient’s left flank.

Later that day the patient was reviewed by the local general surgery team. Abdomen was soft with significant left flank tenderness and oedema but no crepitus. There was also a skin tear and bruising at the left flank (Figure 3).

Given the aetiology of the necrotising fasciitis, the patient was discussed with urology at a tertiary hospital. Discussions centred on whether debridement of necrotising fasciitis should occur at the current treating hospital or whether the patient should be transferred for definitive source control and wound debridement. Given the massive pulmonary embolism and that the patient was relatively stable, decision was made for patient to be transferred for definitive management rather than subject her to two general anaesthetics and procedures. 

The patient was then urgently transferred to a tertiary hospital for ongoing care. On the day of transfer, patient underwent a left open nephrectomy with extensive debridement of left flank and groin. Findings included a large pus-filled retroperitoneal cavity extending to the left gluteal region, an atrophic friable left kidney filled with pus and calculus. Vac dressing was applied to the open wound. The patient was subsequently taken back to theatre the following day for further debridement of necrotising fasciitis. Day 2 post nephrectomy, the patient was again taken back to theatre for a relook and debridement and faeces was found in the abdominal cavity. Upon exploration, two colonic perforations were found near the splenic flexure where the kidney had been removed. An omental patch was performed and a loop ileostomy was formed. After this, a total of five relooks and debridements were performed. Eventually, edges of the flank wound were able to be closed with suture repair but a defect in the central portion of the wound remained. This was closed with a split-thickness skin graft performed by plastic surgery. 

Approximately two months after the initial presentation, the patient was transferred back to the regional hospital for a two-week stint of rehabilitation after which she was discharged home. 

## Discussion

Necrotising fasciitis is a severe soft tissue infection characterised by rapid destruction of tissue, systemic toxicity, and is associated with high rates of morbidity and mortality [1]. It can be classified as either polymicrobial or monomicrobial. Typically, at least one anaerobic species is isolated in combination with Enterobacteriaceae [2]. Diabetes is a particularly important risk factor. Mild emphysematous pyelonephritis can be managed with intravenous antibiotics but severe cases may also require debridement of affected tissues [3]. 

Emphysematous pyelonephritis is a gas-producing necrotising infection involving the renal parenchyma [4]. Onset of symptoms may be abrupt or may evolve slowly. Treatment of life-threatening emphysematous pyelonephritis involves intravenous antibiotics and urgent nephrectomy although studies have reported mild cases can be successfully managed with percutaneous drainage rather than surgery [5]. The prognosis of emphysematous pyelonephritis depends on CT scan findings and has four classification grades (Table 1) [4].

**Table 1:** Huang-Tseng CT classification of emphysematous pyelonephritis.

Class	Characteristics
Class 1	Gas in collecting system only
Class 2	Gas in renal parenchyma without extension to extrarenal space
Class 3A	Extension of gas or abscess to perinephric space
Class 3B	Extension of gas or abscess to pararenal space
Class 4	Bilateral emphysematous pyelonephritis or a solitary functioning kidney with emphysematous pyelonephritis

On reviewing the literature (Pubmed, MEDLINE, and Google Scholar) five case reports which discussed cases of necrotising fasciitis secondary to emphysematous pyelonephritis were found [6-10]. In four of the five cases, the patients were women; all five patients had diabetes mellitus. In terms of causative organism, one case was caused by Citrobacter, one case Klebsiella, two cases E. coli, and one case both E. coli and Klebsiella. 

## Conclusions

There are several learning points from this case report. Firstly, management of DKA proved to be a smokescreen to the underlying pathology and delayed definitive diagnosis and management. The diagnosis of urinary tract infection was made on admission but there was a delay in recognising that the infection may be much more serious than initially thought. In retrospect, a higher index of suspicion for a severe infection was required as an uncomplicated urinary tract infection would be unlikely to cause DKA in a 39-year-old woman without known diabetes. Secondly, this case also reinforces that management of necrotising fasciitis often requires a multidisciplinary approach especially given the primary pathology in this instance. Thirdly, complex cases of necrotising fasciitis such as these are best managed in a tertiary centre where subspecialty services are available onsite. However, it is important to remember that necrotising fasciitis is a surgical emergency and normally necessitates immediate debridement. Thus, surgeons in regional hospitals still play a vital role in management. If this patient was too unstable for transfer, immediate debridement of necrotising fasciitis would have been undertaken with subsequent transfer for ultimate source control. 
